# Analysis and Design of Lattice Structures for Rapid-Investment Casting

**DOI:** 10.3390/ma14174867

**Published:** 2021-08-27

**Authors:** Christopher T. Richard, Tsz-Ho Kwok

**Affiliations:** Department of Mechanical, Industrial and Aerospace Engineering, Concordia University, Montreal, QC H3G 1M8, Canada; richard.chris.cr@gmail.com

**Keywords:** additive manufacturing, rapid-investment casting, lattice structure, castability, digital light processing (DLP)

## Abstract

This paper aims to design lattice structures for rapid-investment casting (RIC), and the goal of the design methodology is to minimize casting defects that are related to the lattice topology. RIC can take full advantage of the unprecedented design freedom provided by AM. Since design for RIC has multiple objectives, we limit our study to lattice structures that already have good printability, i.e., self-supported and open-celled, and improve their castability. To find the relationship between topological features and casting performance, various lattice topologies underwent mold flow simulation, finite element analysis, casting experiments, and grain structure analysis. From the results, the features established to affect casting performance in descending order of importance are relative strut size, joint number, joint valence, and strut angle distribution. The features deemed to have the most significant effect on tensile and shear mechanical performance are strut angle distribution, joint number, and joint valence. The practical application of these findings is the ability to optimize the lattice topology with the end goal of manufacturing complex lattice structures using RIC. These lattice structures can be used to create lightweight components with optimized functionality for various applications such as aerospace and medical.

## 1. Introduction

The emergence and growth of additive manufacturing (AM) has allowed for the use of more complex freeform designs. One promising area of study is cellular lattice structures, which use newer design methodologies. Lattice structures can reduce the amount of material and thus the weight of parts, while maintaining a reasonable strength. Lattice structures can also be used to support complex overhangs, which improves manufacturability [1]. Beyond that, lattice structures show a lot of promise in the biomedical field, e.g., lightweight orthopaedic implants can be fabricated, and the bone in-growth characteristics can be optimized for specific locations within the body [2]. The applications of metallic-AM lattices are widespread, but their manufacturability is limited to a handful or processes. Over the past 30 years, metal-AM has been a topic of interest for many researchers. More specifically, the use of metallic-AM for lightweight cellular structures has been heavily iterated [3]. The result is a vast trove of knowledge regarding materials, parameters, and design methodologies for metallic-AM of cellular lattice structures. However, metallic-AM has some limitations. According to Aboulkair et al. [4], one of the most common defects in selective laser melting (SLM)—a very common metallic-AM process, irrespective of the material, is porosity. Maconachie et al. [5] also mentioned that SLM fabrication of lattice structures is understood to result in manufacturing defects. Moreover, due to the high cost associated with the process, it cannot be used for mass manufacturing of complex parts.

Rapid-investment casting (RIC) is a process used for manufacturing lattice structures. RIC is based on the investment casting (IC), but it relies on AM for pattern making. This allows for RIC to take advantage of the design freedom of AM to produce complex parts. RIC has similar capabilities as metallic-AM. Both metallic-AM and RIC have their respective uses and place in the AM space, but RIC performs better in some applications. For example, RIC does not require sintering, which often leads to considerable shrinkage. More importantly, complex patterns for RIC can be made at a low cost using plastic AM, which opens a door for mass customization of metal parts. To the best of our knowledge, little research has been done to advance the design methodology for RIC of lattice structures when compared to its metallic-AM counterpart. Some of the limited research that has been done includes using fused filament fabrication (FFF) to produce low-cost patterns [6], studying the effect of cross-sectional shape of struts on mechanical properties [7], and finding the optimal filling direction for honeycomb structures [8]. Lattice topology is defined as how the materials and voids are distributed within the unit cell. However, there is no current overarching analysis of the effect that lattice topology has on casting performance and what topological features play the most extensive role in minimizing casting defects. This analysis is required to avoid casting defects in lattice structures, so that they can be faithfully applied to metal parts. Since this paper only focuses on the strut-based lattices, the connectivity and dimension of the struts are used to control the lattice topology.

Given that RIC is a hybrid manufacturing method with both additive and solidification processes, its design considerations stem from both methods. To correctly print a pattern, design for AM needs to be applied. Considerations include print direction, support location, and overhang angle. The quality of the printed pattern depends heavily on the chosen AM method, the topology of the pattern and supports, and the material and process parameters. Design for IC has its own set of considerations: feeding direction, gating/feeding system, and pattern topology. The IC performance depends heavily on the topology of the pattern/gating system and its inherent mold flow. When it comes to the design of lattice structures for RIC, lattice topology is one of the most critical factors that affects printability, castability, and mechanical properties. Different design objectives may have contradicting requirements on the topology, and the research question here is: what lattice topological features have the most significant impact on the overall performance in RIC, and how to improve them? By answering the research question, this paper aims to develop a design methodology for RIC lattice structures. The goal of the design methodology would be to minimize casting defects related to the lattice topology. In this paper, we find a few features, including the relative strut size, joint number, joint valence, and strut angle, significantly affect the casting performance. These features are evaluated using a variety of lattice topologies and structures. Since design for RIC is a multi-objective optimization, we select only the lattice structures with excellent printability, i.e., self-supported and open-celled, and focus on improving the castability without sacrificing mechanical performance. To answer the research question, the lattices will undergo mold flow and mechanical simulation to determine which features are more critical to casting performance. The casting results will be evaluated and compared with the theoretical results to establish design guidelines for RIC lattice structures. These design guidelines are then used to create new designs with casting performance in mind. Finally, a larger scale sample of the best performing design will undergo microscopic void and grain structure analysis to verify the simulation results further. The main objective of this work is to expand the limited research on lattice design for RIC as there is no overarching analysis on the effect of lattice topology on casting performance. The contributions of this paper include:

- A methodology to study the RIC performance of lattice structures is presented to test our hypothesis on the lattice topological features.
- The features are compared and analyzed with the test results, and a set of design guidelines for RIC is created.
- Based on the analysis, new lattice structures are designed and tested for RIC performance.

The paper is organized as follows. The rest of this section briefly reviews the related works. Section 2 details the equipment, materials and methodology used. Section 3 describes the theoretical and experimental results of the study. Section 4 evaluates the results and establishes an order of importance to the topological lattice features for casting performance. Finally, the paper concludes in Section 5.

### Literature Review

Investment casting (IC) was described as taking advantage of a fluid’s ability to assume the shape of its container [9]. IC design is principal to good mold flow [10,11,12]. The most important factor affecting casting defect occurrence, is the quality of the gating system [13]. For example the use of a novel parabolic conical-helical sprue can reduce surface turbulence in metal during mold filling [14]. Less turbulence is important to achieve quality castings. Poor gating system design could also lead to rough surface finish and accuracy, and a design where the size of sprue and runner is unbalanced will produce unstable molten metal flow [15]. Computing and data-driven methods can be used for gating and feeding system design in IC. These methods showed that the gating system’s diameter is most influential on the volume of average shrinkage porosity [16]. Current research is focused on using design optimization in the hopes of reducing the likelihood of casting defects. RIC has similar gating and feeding system design considerations as IC but with the added AM design considerations [17]. These principles can be combined to cast a structurally optimized metal component [18]. The dimensional accuracy of the cast part using RIC is highly pattern dependent [19,20]. Ishida et al. [21] tested the ability of different manufacturing methods to create dimensionally accurate full dental crowns, and they showed that all the methods had their respective drawbacks. The RIC processes, mainly using Stereolithography (SLA) can be found in a review [22].

Presently, the use of lattice structures is desirable because AM has allowed for the fabrication of topologies with great geometrical complexity that was previously unachievable using traditional fabrication techniques [23]. Advancements in lattice structure design and manufacturing include the casting of ordered porous lattice structures with controllable properties [24]. Currently, properties such as high specific stiffness or very high specific strength can only be achieved using geometric methods such as lattice structures. The use of complex lattice structures to achieve specific properties has become more common. For example, Li et al. [25] presented a novel optimization strategy for designing functionally graded cellular structures with desired mechanical properties. Beyond that, lattice structures can be designed using programmable joints resulting in lattices with both stretch- and bending-dominated behavior [26]. These lattice structures with controllable properties can be used in complex applications such as metallic bone design [27]. Current design and manufacturing methods allow for lattices with tailored mechanical properties. This can be achieved by varying the unit cell topology, relative density, and base material. However, there is no existing study on the casting performance of these complex lattices.

## 2. Materials and Methods

This section outlines the parameters and setup used for the theoretical and experimental tests. The characterization methods and lattice structures used in the tests are also detailed.

### 2.1. Materials and Equipment

**Figure 1:**
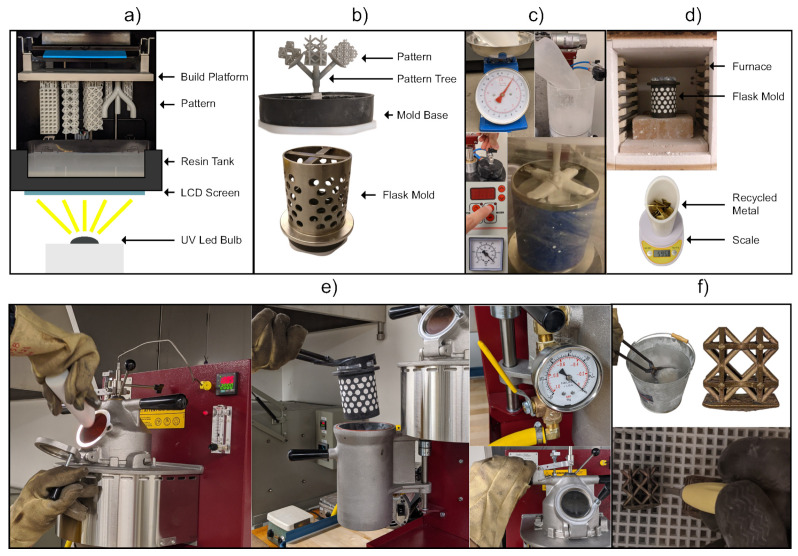
RIC process: (**a**) DLP AM. (**b**) Pattern spruing. (**c**) Plaster mold making. (**d**) Pattern burnout. (**e**) Vacuum casting. (**f**) Post-processing.

The AM machine used in this study is the FabPro 1000 (3D Systems, Rock Hill, SC, USA), a DLP printer with a resolution of 65 microns in the X and Y directions and 30–50 microns in the Z direction (Figure 1a). The material used for this printer is the FabPro Proto GRY (3D Systems, Rock Hill, SC, USA) plastic resin. Proto GRY is a prototyping resin manufactured by 3D systems for prototyping. This resin was used due to its great printability when compared to castable resins. The material composition of the resin is not publicly available. The same manufacturer offers a castable resin. The benefit of castable resin is that it creates less stress and ash in the mold during burnout. However, the castable resin was not rigid enough and did not print well. It also printed a lot slower than the prototyping resin, i.e., four times slower. Although the prototyping resin requires a higher temperature to be burnt out, and the leftover ash could cause some surface defects, it has significantly better printability. The printability of the resin was invaluable as all the lattice topologies tested needed to be self-supporting to ensure there was no need for internal support removal. For this material, the Z direction can achieve a resolution of 50 microns.

The flask used to create the mold is the 4${}^{\prime \prime }$ diameter and 6${}^{\prime \prime }$ tall SuperPerf™ flanged flask (Neutec^®^, Albuquerque, NM, USA), see Figure 1b. A high strength plaster—the Ransom and Randolph Ultra-Vest Maxx—is used as the mold material. The plaster is prepared with the St. Louis 92-4 KG digital vacuum investment mixer (CIMO, Vigevano, PV, Italy), see Figure 1c. Heating of the mold is done in the L17-K12 Furnace (Lucifer, Warrington, PA, USA), see Figure 1d. The casting machine used is the J-2R™ (Neutec^®^, Albuquerque, NM, USA), see Figure 1e. Two casting materials are used in the experiments. One is recycled 70-30 brass with a density of $8.73\times 10^{3}$ g/mm${}^{3}$, and the other is recycled 6061 aluminum with a density of $2.7\times 10^{3}$ g/mm${}^{3}$. Brass is known of with fine details and used for detailed miniatures, sculptures, and jewelry; aluminum is commonly used for lightweight aerospace and automotive applications such as heat exchangers. Since the objective of this paper is to find out the effect of lattice topology on casting performance, these two materials being used is to show that the findings are consistent in materials with quite different properties. The DenPlus Basic Eco Sandblaster is used for post-processing (Figure 1f), and the glass beads used for sandblasting have a size of 50 microns.

### 2.2. Manufacturing Process

The overall RIC process is illustrated in Figure 1, and each step is presented in more details in the following.

#### 2.2.1. Pattern Making

A pattern replicates the shape of the object to be cast, so the computer-aided model (CAD) is used for pattern design. In RIC, the pattern is produced using AM, and we use a DLP printer to make the pattern (Figure 1a). After slicing the CAD model, the pattern is produced via DLP using a projector to cure (solidify) complete layers of liquid resin at a time. The main benefit of DLP is that it can create very high-quality patterns with great surface finishes [28]. Alternate AM processes that can be used for pattern making are multi-jet modeling (MJM), stereolithography apparatus (SLA), and fused filament fabrication (FFF). DLP can achieve more complex overhangs using less supports thanks to it curing layer-by-layer instead of point-by-point and thus has a better self-supporting capability. Additionally, it is a lot faster than MJM and SLA while achieving a comparable resolution. FFF does not produce high enough quality prints for the lattice structure patterns required in this work.

#### 2.2.2. Mold Making and Burnout

**Figure 2:**
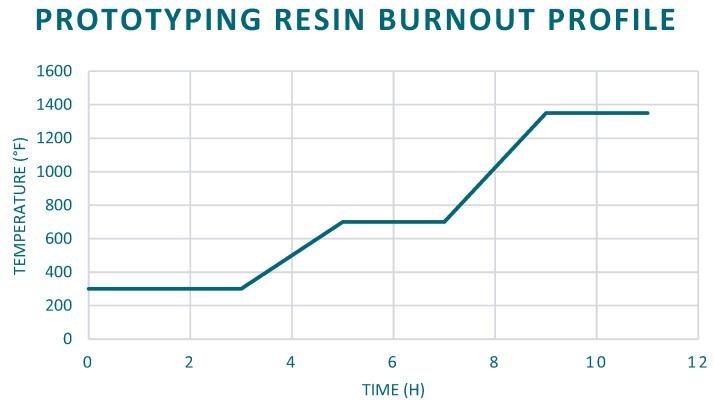
3D Systems FabPro 1000 prototyping resin burnout profile.

To make the mold, the previously printed pattern is placed on a mold base and a steel flask is placed on top of the mold base. The perforated flask is then covered in masking tape to avoid spilling the plaster (Figure 1b). The plaster is then weighed based on the manufacturer’s specifications and the volume of the flask minus the volume of the pattern. The plaster is placed in the mixer, and a vacuum is pulled. Water is then added to the mixer based on the manufacturer’s specifications, and the plaster is hydrated 38%. The mixer then mixes the plaster for 7 min, at which point a knob is pulled to pour the plaster into the mold. Finally, the mold is vibrated to remove any bubbles from the plaster for another 7 min (Figure 1c). The mold is then removed from the mixer and is left for 10 min to dry. The mold containing the resin pattern is then burnt out (Figure 1d) with the custom burnout profile in Figure 2. The burnout profile was modified for the prototyping resin from the castable resin profile. It was deemed to be adequate because it was successfully used to cast patterns of similar shape and volume to the experimental ones with only minor surface defects. Since the patterns will all have the same volume, any defects caused by the resin burnout will be present in all the samples.

#### 2.2.3. Casting

Upon completion of the burnout cycle, the casting machine is preheated to 1038 °C, and the mold is preheated to 538 °C. 150% weight of 70-30 brass was weighed and added to the casting machine’s crucible for melting. The brass weight is calculated based on the density of 70-30 brass and the pattern volume. The reason of 150% mass was chosen is to ensure that there is an excess of metal, eliminating the lack of molten metal as a cause for casting defects. The recycled brass was melted down in the casting machine using argon shielding with a flow of 8 L/min. Once the casting machine hit the melting temperature of 1038 °C, the recycled 70-30 brass was added to the machine, causing the machine’s temperature to drop. Once the temperature rose back to 1038 °C and the metal was molten, the flask was introduced to the flask chamber. Vacuum was then pulled for the flask chamber before pulling the lever that introduces the molten metal to the mold (Figure 1e). The mold was then left under vacuum for 4 min to remove dissolved gases and fill the mold. After 4 min, the vacuum pump was turned off, and the mold was left to cool in the casting machine for 10 min. After 10 min the mold was removed from the machine and left to air cool. The cooling temperatures and times are optimal based on prior testing; these temperatures and time caused the least defects and stress cracking.

For casting experiments involving 6061 aluminum, the whole process is basically the same. The only difference is that the casting machine preheat temperature is 650 °C, and the mold preheat temperature is 315 °C.

#### 2.2.4. Quenching and Post-Processing

Once the mold has cooled to 200 °C from the casting process, the mold can be quenched in a bucket of room temperature water. Most of the plaster dissolves away from the quenching process. After quenching, the remaining plaster caught in small details is sandblasted at 90 Psi (Figure 1f). This removes minimal amounts of material without affecting geometric accuracy. Once all the plaster is removed from the sample, the feeder is removed using a fret saw.

### 2.3. Lattice Designs

To test the casting performance of lattice structures, two sets of experiments with different designs are conducted. Two materials are used for the experiments to ensure it is the design rather than the material affecting the performance.

#### 2.3.1. Set 1

**Figure 3:**
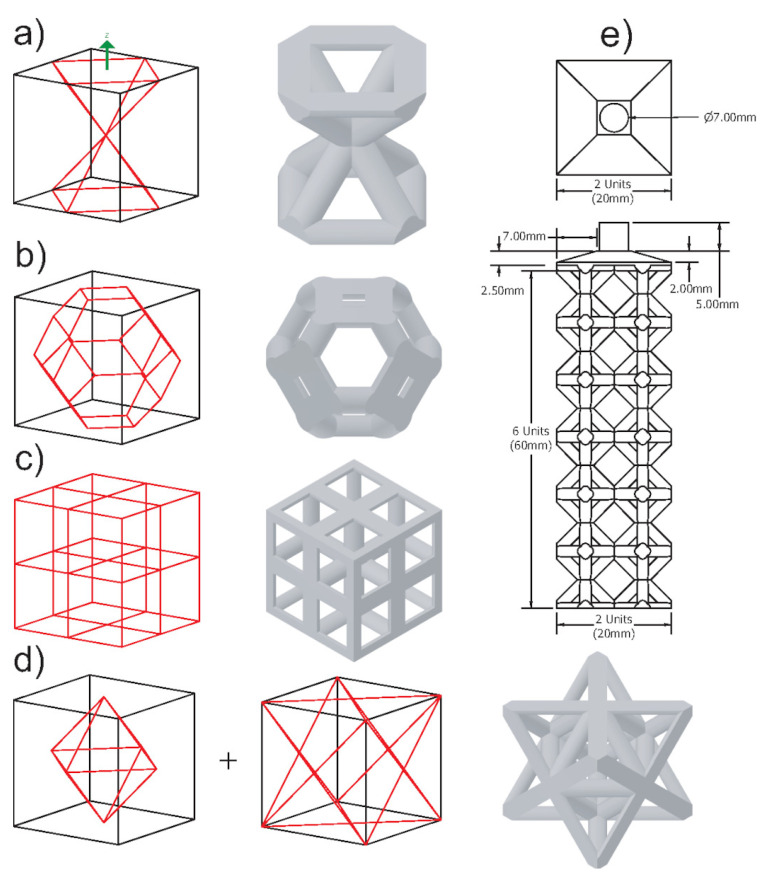
Test 1 lattice cells and structure: (**a**) Rhombic. (**b**) Kelvin Cell. (**c**) Cubic. (**d**) Octet-Truss. (**e**) 2×2×6 structure.

In the first experiment, the set of lattice topologies includes rhombic, kelvin cell, cubic, and octet-truss, as shown in Figure 3a–d. These topologies were chosen as they are commonly used structures in AM, and they vary a lot in topology while remaining open-celled and self-supporting. Each lattice unit cell is a 10 mm × 10 mm × 10 mm cube, and the green arrow on the wireframe cells is the Z-axis, which is the filling direction. Their connectivity is shown to better understand how they connect in 3D. All the topologies have a straight circular strut cross-section and a cubic packing strategy. The scope of topologies was narrowed to limit the number of possible designs. All four topologies have a constant volume, and the strut size is changed to achieve this. The density of the unit cells is kept constant at 20%, and they are designed using Autodesk Inventor. The strut sizes for these topologies range from 1.373 mm to 1.980 mm. To better grade their filling performance and proneness to casting defects, the structures to be cast contain 2×2×6 unit cells as shown in Figure 3e, and they were all fed via a 7 mm cylindrical feeder.

#### 2.3.2. Set 2

**Figure 4:**
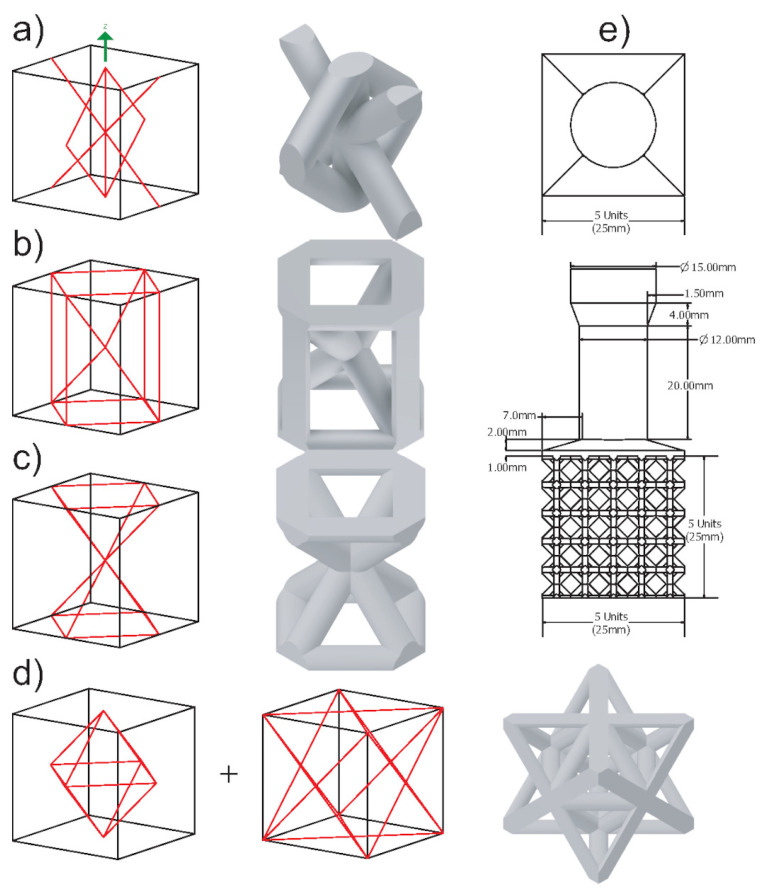
Test 2 lattice cells and structure: (**a**) Proposed cell. (**b**) Hourglass. (**c**) Rhombic. (**d**) Octet-Truss. (**e**) 5×5×5 structure.

For the second experiment, the main goal is to use the first experiment’s observations to create lattice topologies better for casting performance. Rhombic and octet-truss are being passed along from the previous experiment to serve as benchmarks compared to the previous experiment. Along with those two topologies, two more have been designed. The four unit cells are the proposed cell, hourglass, rhombic, and octet-truss. The topologies, as well as their overall dimensions and orientation, can be seen in Figure 4. The proposed cell was designed purely for good casting performance. It has a low number of joints, low joint valence (number of struts at a joint), most of its struts are 45° and it has a large relative strut size. The hypothesis is that this combination of features will lead to a better casting performance. The hourglass structure has a balance of vertical, horizontal, and 45° struts. This structure is used to evaluate further the effect of strut angle distribution on casting performance.

The unit cell size for this experiment is 5 mm × 5 mm × 5 mm. This was chosen to observe more casting defects without exceeding the dimensions of the flask. The casting material was changed to 6061 aluminum for this set. Due to the higher solidification shrinkage of aluminum compared to the brass, set 2 is a more challenging test beyond the geometry setting. This too will contribute to the ability to observe more casting defects to better grade the lattice topologies. The strut sizes for these samples ranged from 0.686 mm to 1.111 mm. This finer strut size will increase the likelihood of premature melt solidification which will more significantly differentiate the different topologies’ performance. The structures to be cast contain 5 × 5 × 5 unit cells as shown in Figure 4e. To feed the larger number of unit cells, the feeder size is being increased to 12 mm. A 15 mm opening is also used to interface with the sprue base for the mold.

### 2.4. Characterization

Analyses and characterizations based on both computer-aided engineering and physical experiments are conducted. They are detailed here.

#### 2.4.1. Mold Flow Simulation

Mold flow simulations on various lattice structures were performed to grade the structures based on their casting performance. These simulations were performed in Altair Inspire Cast. The simulations were not used as exact representations of the different lattice structures’ mold flow but were used as a comparison tool to see which samples performed better based on the following criteria: filling time, porosity, and cold shuts. Cold shuts refer to when multiple joining metal flows cool before properly fusing together. The lattice structure geometry for the mold flow simulations is the same as the one to be cast in Figure 3e. Since the complexity of the second experimental structures increased based on the results of the first. They were too complicated to simulate on a standard PC, and thus this simulation was not conducted (only physical experiments).

The mold flow simulation was performed using the parameters listed in Table 1. The simulation was a gravity process with the fill parameter set as a constant liquid level on the sprue. The casting method is investment casting, and a shell mold was chosen to simplify the simulation. The shell thickness of 50 mm is quite large and comparable to the casting experiments’ flask mold. The 7 mm cylindrical feeder fed the molten metal as in Figure 3e. The elements used by the mold flow simulations are tetrahedral, and the software chose their size.

#### 2.4.2. Mechanical Finite Element Analysis

Mechanical simulations were performed using Ansys Workbench 19.2 (Ansys, Canonsburg, PA, USA). The material properties used for the simulations were 70-30 brass found in Table 2.

**Figure 5:**
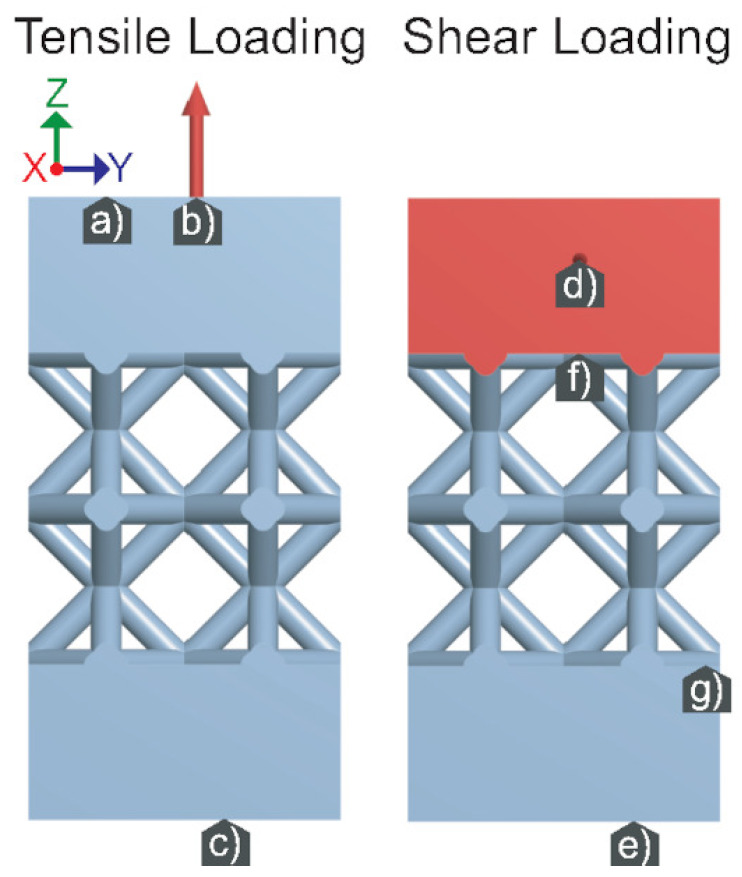
Tensile loading conditions: (**a**) Displacement allowed only in Z. (**b**) Applied force. (**c**) Fixed support. Shear loading conditions: (**d**) Applied force. (**e**) Fixed support. (**f**,**g**) Displacement allowed only in X and Y.

The geometry for this simulation was simplified as shown in Figure 5.

This was done to save on computation time and have a better visualization since the results were negligibly different from the simulations with the more complex geometry. Two static structural, mechanical simulations were performed per lattice sample: tension and shear. For the lattice topologies observed, the focus was lightweight, rigid topologies. Therefore, the topologies were only loaded right up till the onset of plastic deformation. The applied load was stopped before the max principal stress exceeded either the yield or shear strength. Additionally, two large blocks of material were added to both ends of the lattice structure to ensure the loads are applied uniformly to the structure. A fixed support was also added to the bottom face, which restricts movement in every direction. For tensile loading, a upward 15 kN force was applied to the top face. For shear loading, a horizontal 15 kN force was applied to the front face.

#### 2.4.3. Microscopic Analysis

The best performing lattice topology will be 3D printed, molded, and cast at a larger scale. The sample will then be cut into three smaller samples. The first sample will represent a joint of valence two, the second one of four valence, and the last one six valence. Two sets of these six samples will be prepared. To perform grain structure analysis on the castings, samples are cut using a fret saw. Next, they are mounted in Bakelite using the Bueller SimpliMet 3000 compression press. The samples are lapped using 300, 400, 600, 800, and 1200 grit sandpapers. The samples are mirror-polished using alumina powder. Finally, they are etched using 200 mL distilled water, 10 gm Ferric chloride, and a 50 mL Hydrochloric acid solution. The void and grain structure analyses are done using the VHX-6000 digital microscope (Keyence, Osaka, Japan) and ImageJ (NIH, Bethesda, MD, USA). Using the microscopic analysis images, each sample’s void ratio can be determined, and the grain structure can be visualized. Although it is commonly known that porosity will increase with joint valence, the result should be an understanding of what valence number is acceptable in RIC lattice structures.

## 3. Results

### 3.1. Mold Flow

**Figure 6:**
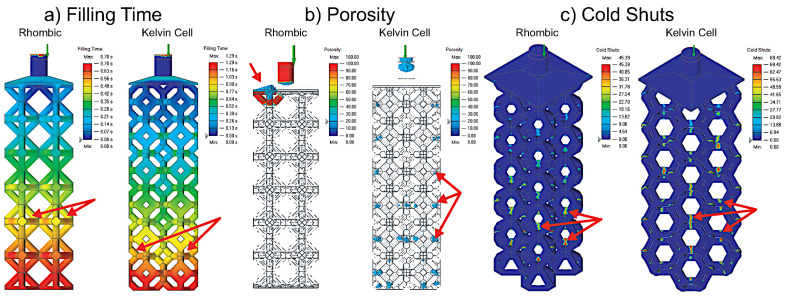
Flow simulation results for rhombic and kelvin cell.

The results of the mold flow simulation can be seen in Figure 6. The 3D plots of only the rhombic and kelvin cell structures are presented because both the cubic and octet-truss samples solidified before filling according to the simulation, so their results were not plotted by the software. The three observed casting properties that showed the largest deviation across the lattice topologies were filling time, porosity, and cold shuts. First, from the comparison of filling time, we can see that the rhombic structure fills the fastest, and the filling time does not differ much in the x and y direction but only in the filling direction. Secondly, the regions with 20% porosity were chosen to be unacceptable and highlighted. The kelvin cell structure has repeatable porosity located at the horizontal struts (90° from filling direction). The rhombic structure shows almost no porosity of 20% in the body, but just at the top. This shows a highly directional filling, which is desirable in casting. Finally, the likelihood of cold shuts were observed. The magnitude of cold shuts in the kelvin cell sample is higher than that of the rhombic sample. The high potential formation of cold shuts for these two structures were consistently located on horizontal struts (90° from filling direction).

### 3.2. Cast 1

**Figure 7:**
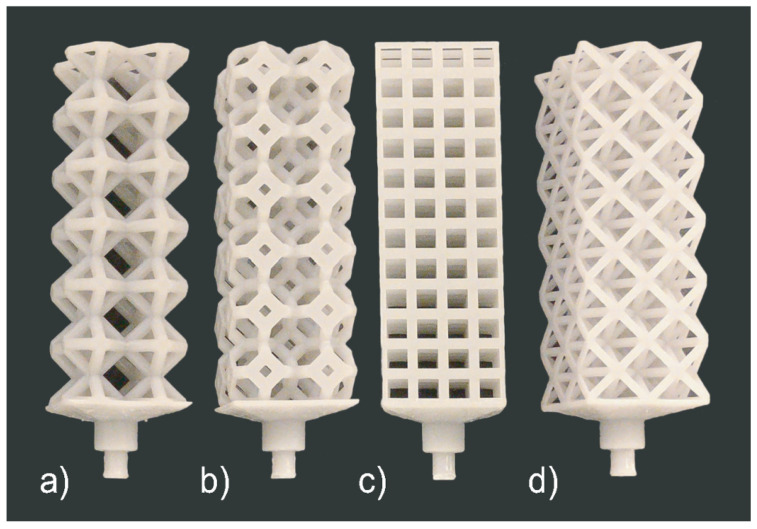
Set 1 printed patterns: (**a**) Rhombic, (**b**) Kelvin Cell, (**c**) Cubic, and (**d**) Octet-Truss.

**Figure 8:**
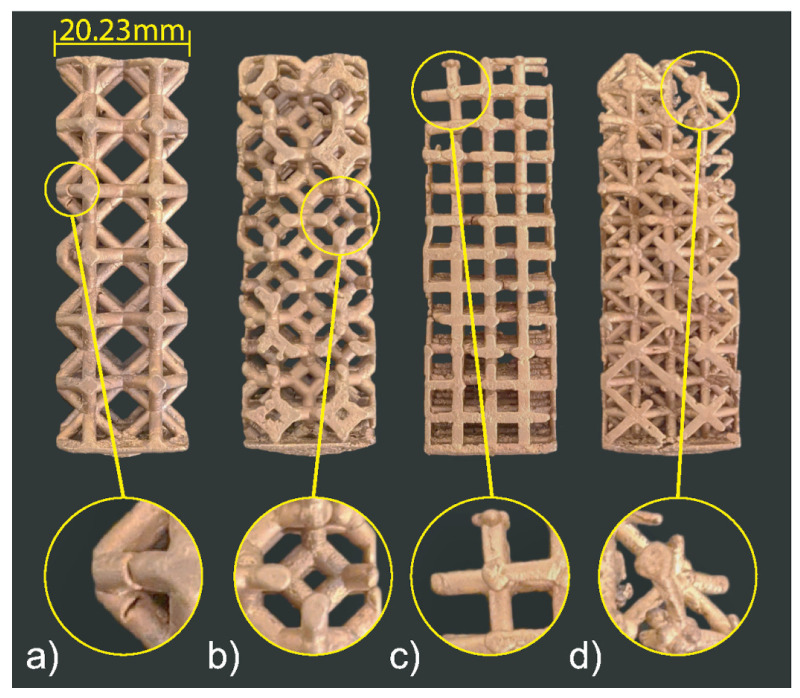
Set 1 cast structures: (**a**) Rhombic, (**b**) Kelvin Cell, (**c**) Cubic, and (**d**) Octet-Truss.

Following the presented manufacturing pipeline, all the lattice structures were successfully 3D printed without supports (see Figure 7). The patterns are then molded and cast, and the final casts can be seen in Figure 8. Through visual inspection, we can observe the location and severity of casting defects. The octet-truss and cubic structures did not fill, contrarily the rhombic and kelvin cell did. The kelvin cell has consistent defects on its horizontal struts (90° from filling direction). The rhombic structure also has defects on a few horizontal and diagonal struts, but this was not a consistent defect. Furthermore, the weights and dimensions of the CAD models, casts, and printed patterns are listed in Table 3. The measurements were taken using a caliper at three points on the structure’s width and the struts. These three measurements are averaged, and they were taken randomly on the samples. For the width, any unfilled portions were avoided to make the measurements more accurate. Let it be noted that all the printed lattice structures were around 1% larger in structure width than the CAD geometries. The biggest contributor to the inaccuracy in size seems to be caused by the 3D printing process, but this could be accounted for and corrected in the CAD models or the printer software. Because some samples filled more than others, the measurements may not be a perfect representation as the unfilled samples will have a more significant variation in dimensions. The percent fill was calculated by comparing the cast part’s mass from the CAD model to what it is. The percentage fill is based on the weight of the cast samples with the feeder removed compared to the CAD model’s weight. According to the percent fill, the rhombic structure filled the most, followed by the kelvin cell, cubic, and octet-truss. The percentage fill and visual inspection agree. Overall, the rhombic structure has the best casting performance. The structure seems to have flowed well based on the number of defects. The casting performance from best to worst was rhombic, kelvin cell, cubic, and octet-truss.

### 3.3. Cast 2

**Figure 9:**
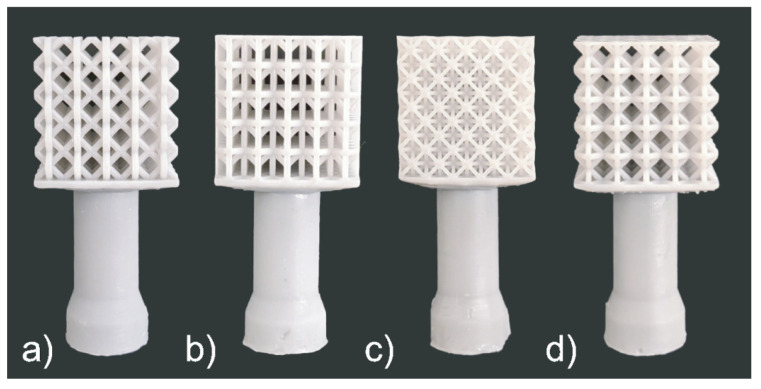
Set 2 printed patterns: (**a**) Proposed cell, (**b**) Hourglass, (**c**) Rhombic, and (**d**) Octet-Truss.

**Figure 10:**
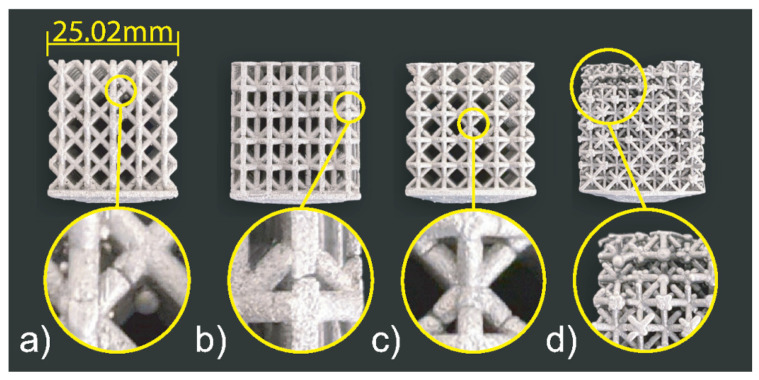
Set 2 cast structures: (**a**) Proposed cell, (**b**) Hourglass, (**c**) Rhombic, and (**d**) Octet-Truss.

All the four lattice structures in the second set are also successfully printed without supports, molded, and cast, as shown in Figure 9 and Figure 10. There was no significant variation in the samples’ weight (see Table 4). According to the percent fill, although the difference of the top-three was low, the most successful structure was the hourglass proceeded by the proposed cell, rhombic, and finally the octet-truss. However, from visual inspection, it is clear that the proposed structure showed the fewest visible defects, followed by the hourglass, rhombic, and finally octet-truss. The proposed cell sample only had one visibly unfilled strut. The hourglass sample had many visible defects present in its vertical (0° struts) as well as its joints among the vertical (0°), horizontal (90°) and diagonal (45°) struts. The rhombic structure had many defects similar to the first casting experiment with voids at the perpendicular (90°) struts and the high joint valence joints. Finally, the octet-truss sample did not fill. From these results, we can see that the two new structures designed based on the observation from experiment 1 have better casting performance.

### 3.4. Mechanical Properties

**Figure 11:**
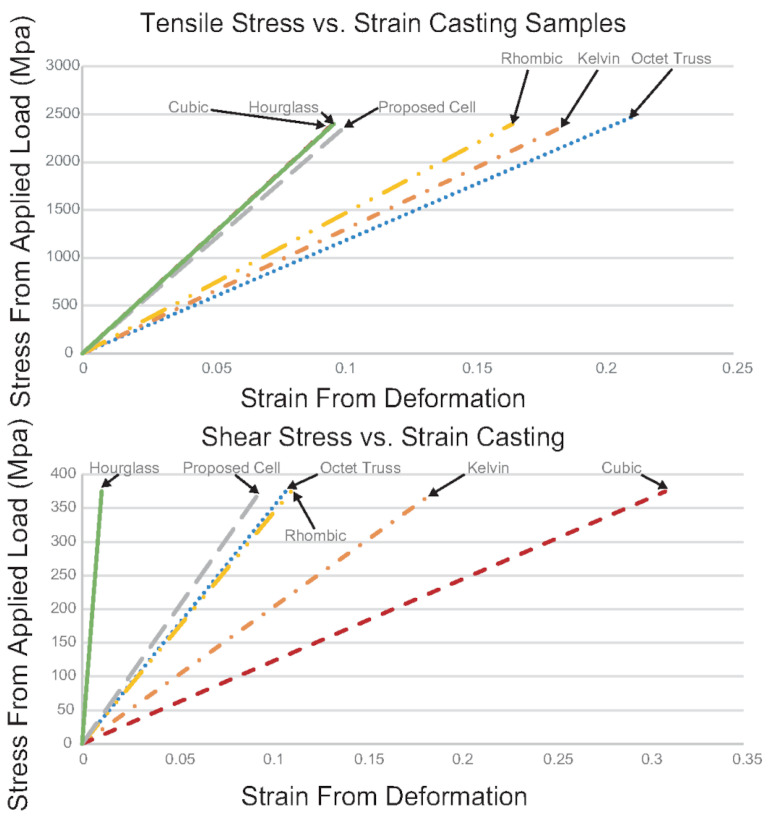
The tensile and shear stress-strain curves from FEA results.

The tensile stress-strain curve up until plastic deformation can be seen in Figure 11. The kelvin cell, cubic, proposed cell, and hourglass structures were unloaded before exceeding the yield strength. The rhombic and octet-truss structures were unloaded before exceeding the shear strength. From the graph, it can be seen that under tensile loading, the hourglass, proposed cell and cubic samples performed the best. These three topologies had the highest equivalent tensile modulus (see Table 5). The rigidity of the structures under tensile loading from highest to lowest is cubic, hourglass, proposed cell, rhombic, kelvin, and octet-truss. Similarly, the line’s slope for shear stress against strain tells how much deformation occurs for a given applied shear load. The equivalent shear modulus of the structures can also be seen in Table 5. The structure’s rigidity under shear loading from highest to lowest is hourglass, proposed cell, octet-truss, rhombic, kelvin, and cubic. In terms of overall mechanical performance for shear and tensile, the hourglass structure performed the best closely followed by the proposed cell. The rhombic structure was the only other structure that performed well for both loading conditions. None of the other structures performed well for both loading conditions. Let it be noted that all the mechanical properties are obtained from FEA. This is because most fabricated samples have incomplete fills, and they cannot be tested and compared. Although the mechanical analyses here are not physically validated, since the objective of this work is to find out the effect of lattice topology on casting performance considering the mechanical properties, they are meaningful enough to this study.

### 3.5. Voids and Grain Structures

**Figure 12:**
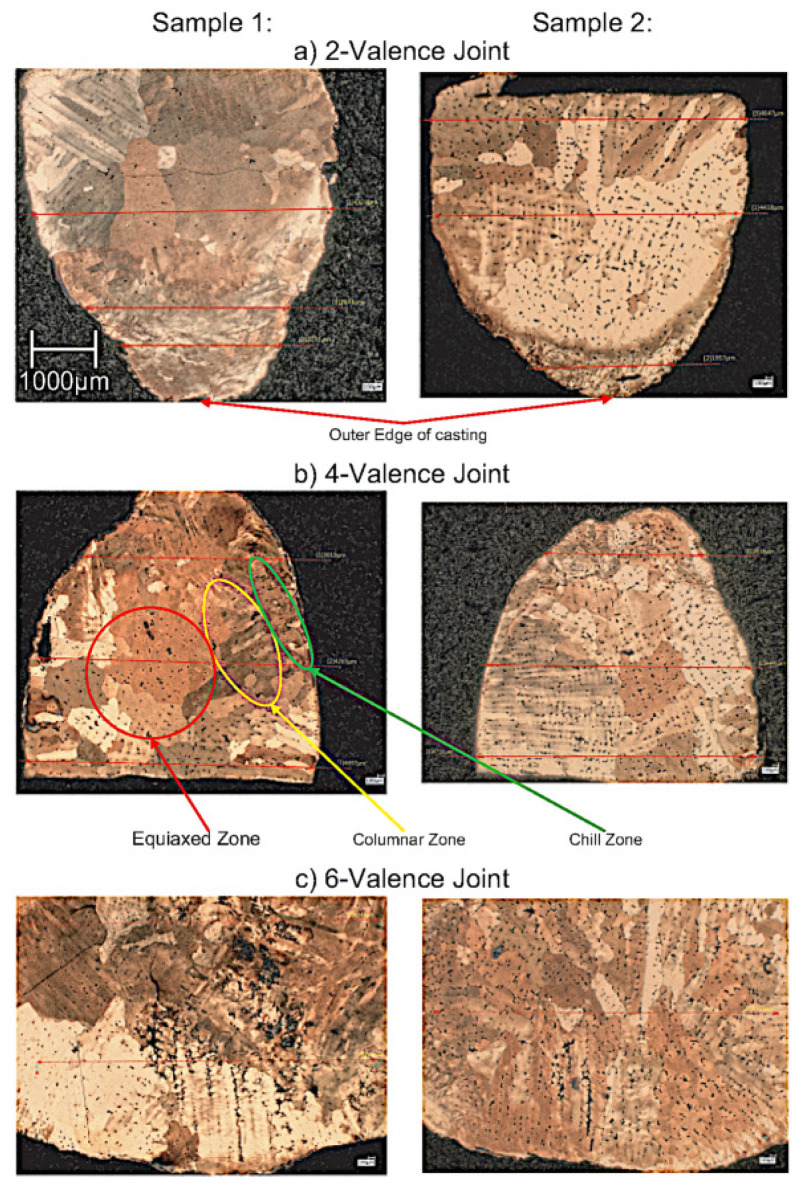
Grain structure analysis using optical microscopy. Images 50× magnification.

In casting, it is known that different grain structures would be produced during solidification depending on how heat is removed. The most preferable structure is the one with spherical randomly oriented crystals because it has isotropic properties (uniform in all directions). Therefore, the geometry should be designed such that the result has the preferable structure as much as possible. This is one of the important factors in measuring casting performance, and thus a grain structure analysis is conducted to find out the effect of joint valence (lattice topology) on grain structure formation (casting performance). Following the procedure in Section 2.4.3, the most successful topology—the proposed cell, is cast again at a larger scale to study the voids and grain structures. The cast sample is cut on a plane containing 2-valence, 4-valence, and 6-valence joints. The valence of a joint refers to how many struts connect at the joint. The results of the microscopic analysis can be broken down into two parts. The first is the analysis of resultant grain structure, and the second is the analysis of porosity in the cast samples. The resultant grain structure for the 2-valence, 4-valence, and 6-valence joints can be seen in Figure 12. The 2-valence and 4-valence joints both show clear indications of a chill, columnar, and equiaxed zone. The 6-valence joint seems to lack a fully formed equiaxed zone. This is most likely due to the cooling rate of this joint. The grain area distribution was not determinable due to voids limiting the ability for software to determine the grain boundaries.

**Figure 13:**
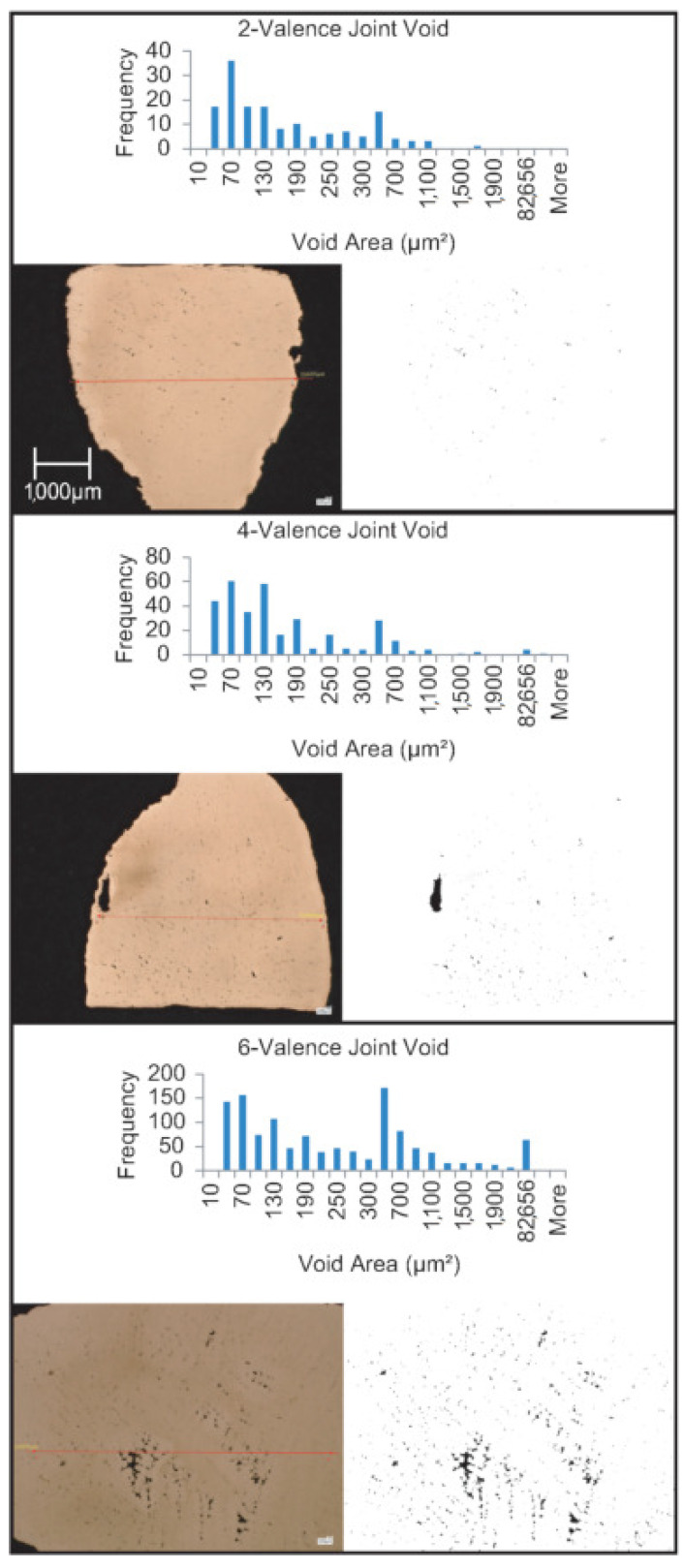
Microscopic analysis for porosity using optical microscopy. Images 50× magnification.

Thick casting sections caused by intersections—a.k.a. hot spots—can result in localized shrinkage. This localized shrinkage results in voids and porosity. In principle, the larger the valence number, the more voids. It is important to understand the acceptable extent. The porosity results can be seen in Figure 13, which shows the microscopic images at 50× magnification without having etched the sample. In this study, multiple images were analyzed, and the results were repeatable. The area and frequency of each void were tabulated. The results for void ratio, number of voids, and max/min void area are summarized in Table 6. We see a clear trend from the results. With the increase in the joint valence, a higher void ratio is observed. The void ratio increases from 0.21% in the 2-valence joint to 1.45% in the 4-valence joint and finally to 3.35% in the 6-valence joint. The void ratio is based on the sum of the void area, so the void area shows the same trend. The number of defects also increases with the increase in joint valence. The only outlier in terms of behavior is the max void size. The max is higher for the 4-valence joint than the 6-valence joint. The large defect in the 4-valence joint is quite close to the strut’s surface and could be a surface defect when looking at the structure as a whole. Regardless of this outlier, the overall void ratio still ends up being lower than the 6-valence one. These results show that it is indeed the higher the valence number, the worse the casting performance. However, equiaxed zones can be seen in all these cases. Equiaxed grains are more desirable as they exhibit better mechanical performance. Therefore, the joint valence of 6 is still acceptable.

## 4. Discussion

**Figure 14:**
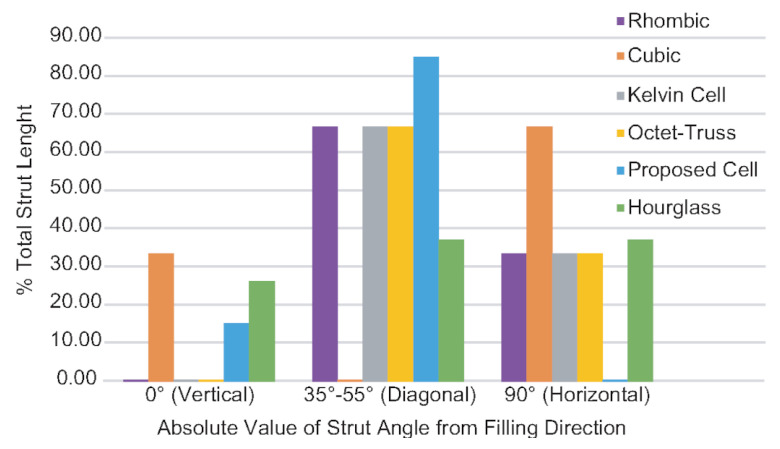
Topology strut angle distribution.

From all the results: mold flow, the two casting experiments, and the FEA, we have narrowed down the factors that significantly affect casting performance. The features that play a role in the success of the cast lattice topologies are relative strut size, number of joints, joint valence, and strut angle distribution. The casting experiments’ results and the overall performance of each lattice topology can be explained using these features. The relative strut size is the strut size divided by the unit cell width, which is a property that will remain the same regardless of unit cell width. The number of joints refers to the total number of points within the cell where struts connect. Joint valence refers to how many struts connect at a given joint. The above features are listed in Table 7 for each lattice topology tested. Strut length can be measured for different strut angles and divided by the total to determine the strut angle distribution (see Figure 14).

When analyzing the casting and mold flow results, the effect of relative strut size can be seen most significantly in the cubic and octet-truss topologies. The two structures have lower relative strut sizes of 0.16 and 0.14 when compared to the more successful proposed and hourglass topologies of 0.22 and 0.20 (see Table 7). Lower relative strut sizes result in slower filling times which often results in premature melt solidification. Therefore, it is clear that the relative strut size plays the largest role in the success of a lattice structure casting. This ratio gives an idea of the negative effect that adding more struts for rigidity has on the strut size and in turn the metal flow. It allows us to determine which structures create an efficient short path for the metal to flow through.

Next in the level of importance is the number of joints. From the mold flow and experimental castings, it is clear that the topologies with the highest number of joints performed very poorly. The effect can be seen in the cubic (27), kelvin cell (24) and octet-truss (14) topologies. The remaining three topologies with a joint number of 9 all performed well. To add a level of granularity to the analysis, these three topologies can be further classified based on their joint valence. The proposed structure performed better than the other two due to its low joint valence (4.58), this trend continues for the hourglass (5.74) and then the rhombic (8). This behavior can be further supported by the void analysis results. A higher void ratio can be observed as the joint valence is increased from 2 to 4 to 6.

Finally, the effect of strut angle can definitely be seen in the casting results and the mold flow results, with defects often occurring at vertical or horizontal struts. Regardless, strut angle seems to have the smallest effect on the performance. The hourglass topology illustrates this as it was the second-best performant but it has a balance of vertical, diagonal, and horizontal struts. This topology would have performed worse due to its horizontal and vertical struts if strut angle had more of an impact on the performance. From the FEA results, the presence of horizontal, diagonal, and vertical struts is important for mechanical strength under tensile and shear. Some of the structures were tested by Després et al. [29]. They observed similar behavior as was observed in our FEA results. The diagonal struts contribute to the shear performance whereas the vertical struts contribute to the bending, tension and compression performance.

Overall, looking at a balance of casting performance and mechanical strength, it appears that the best way to improve casting performance is optimizing for relative strut size, number of joints, and joint valence. Mechanical performance can be achieved through strut angle distribution.

### Design Guidelines

To better establish and understand the design guidelines for RIC, the performance grading of all the topologies can be seen in Table 8. The topologies are graded from 1 to 4, where 4 is the best and 1 is the worst performance. This table also includes the tensile and shear performance for reference. The topological features are listed in decreasing order of casting performance from left to right. The two topologies (proposed and hourglass) performed the best experimentally, as shown in the table. They scored at least 3 across the board and scored 4 in the most important categories (relative strut size, number of joints, and joint valence). The mechanical performance was also 4 due to the angle distribution, which includes vertical, horizontal, and diagonal struts [29]. In addition, the number of joints and joint valence affect mechanical strength. This effect was observed in the FEA results and is supported by Li et al. [30]. The authors stated that lattice cells’ deformation mode changes from bending-dominated to stretch-dominated with the increase of the joint valence. Stretch-dominated is the more rigid one of the two behaviors. Although rigidity can be achieved without a high number of joints and joint valence, as Maxwell’s criterion of rigidity is limited as demonstrated by Chen et al. [31]: if FEA shows a rigid lattice, then the net lattice is rigid.

From these findings and analyzes, a set of design guidelines for RIC can be drawn. On top of the design considerations for IC (e.g., minimum strut size of 1 mm) and DLP AM (e.g., open-celled). The design guidelines for RIC in order of importance are as follows:

- The relative strut size should be kept below 0.2.
- The number of joints should be kept below 9.
- The max and mean joint valence of 8 or less is recommended.
- For mechanical performance, the strut angle distribution should include vertical, diagonal, and horizontal struts.

Let it be noted that a similar condition as in this paper should be met for faithful use of the design guidelines. Besides the machines and materials used, they include straight and circular cross-sectioned struts, cubic packing strategy, and the size of unit cell ranging from 5 mm to 10 mm. By far from what has been tested, the proposed, hourglass and rhombic topologies meet all these criteria and achieve good casting and mechanical performance.

## 5. Conclusions

In summary, it is clear that as with other metallic-AM processes, RIC can take full advantage of AM’s unprecedented design freedom. RIC was successfully used to create a variety of lattice structures. These structures were used to determine the topological lattice features critical to casting performance. From those results, a methodology to study the performance of RIC lattice structures has been established. In this methodology, topological features are compared and analyzed using the test results. This analysis results in a set of design guidelines for RIC. The features established to affect casting performance in descending order of importance are relative strut size, number of joints, joint valence, and strut angle distribution. Without controlling these features, hot spots, porosity and premature solidification can occur. All four proposed topological lattice features directly affect a lattice’s feed paths and flow velocity/connectivity. Although the design guidelines share the same logic as the ones for general casting, they differ in that they are specifically tailored towards optimizing cellular structures. Cellular structures by nature have a high potential for flow restriction or high connectivity. For this reason, more targeted design guidelines than the general casting ones are needed. The features deemed to have the most significant effect on tensile and shear mechanical performance are strut angle distribution, number of joints, and joint valence. With the design methodology, the proposed cell and hourglass topologies were created. These lattice topologies had the best overall casting and mechanical performance of all the tested lattices. The limitations of the current work include testing only strut-based lattice topologies. Future work could expand beyond this. Future work could also further refine the design methodology and automate the design process using software-driven methods.

## Figures and Tables

**Table 1:** Mold flow Altair Inspire Cast 2019.3 material properties.

Property	Value	Unit
Material	CW505L Brass	N/A
Molding Material	Plaster	N/A
Melting Temp.	1093	°C
Preheat Temp.	538	°C
Shell Mold Thickness	50	mm

**Table 2:** Ansys 70/30 [Cu/Zn] brass material properties.

Property	Value	Unit
Density	8530	kg/m^3^
Young’s Modulus	$10\times 10^{10}$	Pa
Poisson’s Ratio	0.331	
Bulk Modulus	$8.08678\times 10^{10}$	Pa
Shear Modulus	$3.080390\times 10^{10}$	Pa

**Table 3:** Lattice topology comparison casting experiment 1.

	**Rhombic**			**Kelvin Cell**		
	**Mass (g)**	**Cell Width (mm)**	**Strut Diameter (mm)**	**Mass (g)**	**Cell Width (mm)**	**Strut Diameter (mm)**
CAD	47.162	20.00	1.980	47.163	20.00	1.976
Printed	N/A	20.23	2.013	N/A	20.21	2.090
Cast	49.000	20.19	2.077	45.840	20.15	2.047
% Fill	103.90			97.19		
	**Cubic**			**Octet-Truss**		
	**Mass (g)**	**Cell Width (mm)**	**Strut Diameter (mm)**	**Mass (g)**	**Cell Width (mm)**	**Strut Diameter (mm)**
CAD	47.142	20.00	1.629	47.163	20.00	1.373
Printed	N/A	20.20	1.723	N/A	20.08	1.447
Cast	40.350	20.15	1.693	38.500	20.11	1.417
% Fill	85.59			81.63		

**Table 4:** Lattice topology comparison casting experiment 2.

	**Hourglass**			**Proposed Cell**		
	**Mass (g)**	**Cell Width (mm)**	**Strut Diameter (mm)**	**Mass (g)**	**Cell Width (mm)**	**Strut Diameter (mm)**
CAD	12.005	25.00	1.022	12.004	25.00	1.111
Printed	N/A	25.19	1.013	N/A	25.02	1.147
Cast	13.030	25.01	1.157	12.750	24.93	1.113
% Fill	108.54			106.21		
	**Rhombic**			**Octet-Truss**		
	**Mass (g)**	**Cell Width (mm)**	**Strut Diameter (mm)**	**Mass (g)**	**Cell Width (mm)**	**Strut Diameter (mm)**
CAD	12.005	25.00	0.989	12.005	25.00	0.686
Printed	N/A	25.18	1.028	N/A	25.10	0.743
Cast	12.540	24.86	1.003	11.740	20.08	1.447
% Fill	104.46			97.79		

**Table 5:** Geometric stiffness for simulated Ansys lattice structures. $E_{\mathrm{eq}}$ is the Equivalent Tensile Modulus and $G_{\mathrm{eq}}$ is the Equivalent Shear Modulus.

Topology	$E_{\mathrm{eq}}$ (Mpa)	$G_{\mathrm{eq}}$ (Mpa)
Rhombic	14,528	3387.3
Kelvin	12,849	2005.9
Cubic	24,941	1214.8
Octet-Truss	11,710	3478.2
Proposed Cell	23,501	3978.4
Hourglass	24,847	36,225.0

**Table 6:** Void properties. All areas are in μm^2^.

	2-Valence	4-Valence	6-Valence
Sum of void area	28,946	199,025	786,608
Max void area	1555	129,721	82,656
Min void area	20	20	20
Total void #	154	326	1209
Total strut area	13,579,304	13,729,209	23,476,110
Void ratio (%)	0.21	1.45	3.35

**Table 7:** Lattice topology strut size and joint characteristics.

	Proposed	Hourglass	Rhombic	Cubic	Kelvin Cell	Octet-Truss
Relative Strut Size	0.22	0.20	0.20	0.16	0.20	0.14
Number of Joints	9	9	9	27	24	14
Max Joint Valence	6	6	8	6	4	16
Min Joint Valence	4	4	8	6	4	16
Mean Joint Valence	4.58	5.74	8	6	4	16

**Table 8:** Lattice topology performance grading. 4: very good, 3: good, 2: fair, 1: poor.

	Proposed	Hourglass	Rhombic	Kelvin	Cubic	Octet-Truss
Relative Strut Size	4	4	3	3	2	1
Number of Joints	4	4	4	1	1	2
Joint Valence	3	3	3	4	3	1
Strut Angle Distribution	4	3	3	3	2	3
Tensile	4	4	3	3	4	2
Shear	4	4	4	2	1	4

## Data Availability

Publicly available datasets were analyzed in this study. This data can be found here: https://bit.ly/RICData (accessed on 22 August 2021).
